# American mitogenome reference for the tropical brown dog tick, *Rhipicephalus linnaei* (Audouin, 1826)

**DOI:** 10.1016/j.crpvbd.2024.100194

**Published:** 2024-06-18

**Authors:** Consuelo Almazán, Lorena Torres Rodríguez, Abdullah D. Alanazi, Jan Šlapeta

**Affiliations:** a Immunology and Vaccines Laboratory, Facultad de Ciencias Naturales, Universidad Autónoma de Querétaro, Querétaro, Qro., 76140, Mexico; b Facultad de Medicina Veterinaria y Zootecnia, Universidad Autónoma de Tamaulipas, Km 5 Carretera Victoria-Mante, Cd. Victoria, Tam., CP, 87000, Mexico; c Department of Biological Sciences, Faculty of Science and Humanities, Shaqra University, P.O. Box 1040, Ad-Dawadimi, 11911, Saudi Arabia; d Sydney School of Veterinary Science, Faculty of Science, The University of Sydney, Sydney, New South Wales, 2006, Australia; e Sydney Infectious Diseases Institute, The University of Sydney, New South Wales, 2006, Australia

**Keywords:** *Rhipicephalus sanguineus* complex, mtDNA, Brown dog tick, Phylogeny, Genome, Next-generation sequencing

## Abstract

The brown dog tick, *Rhipicephalus linnaei* (Audouin, 1826), is distributed across the American continent and is formerly known as the “tropical lineage”. It belongs to the *Rhipicephalus sanguineus* (Latreille, 1806) species complex, referred to as *R. sanguineus* (*sensu lato*). Mitochondrial genome sequences are frequently used for the identification and represent reference material for field studies. In the present study, the entire mitochondrial genomes of *R. linnaei* (∼15 kb) collected from dogs in Mexico were sequenced and compared with available mitogenomes of *R. sanguineus* (*s.l*.). The mitochondrial genome is ∼90% identical to the reference genome of *R. sanguineus* (*sensu stricto*, former “temperate lineage”) and > 99% identical to *R. linnaei* mitogenome derived from the neotype. Two additional mitogenomes were obtained and described as *R. linnaei* and *R. turanicus* from dogs in Saudi Arabia. The present study delivers a molecular reference for *R. linnaei* from America and complements *R. linnaei* mitogenomes from Africa, Asia and Australia. We propose to consider the complete mitogenome, as the reference for American *R. linnaei*, even when partial mitochondrial *cox*1, *12S* rRNA or *16S* rRNA genes are characterised.

## Highlights

• Mitogenome and phylogenetic analysis of *Rhipicephalus linnaei* from Mexico.
• Mitogenomes robustly resolve the conspecificity of *Rhipicephalus linnaei* across the tropics.
• Mitogenomes from Mexico and Saudi Arabia resolve the phylogeny of the *Rh. sanguineus* (*s.l.*) species complex.

## Graphical abstract

**Figure:**
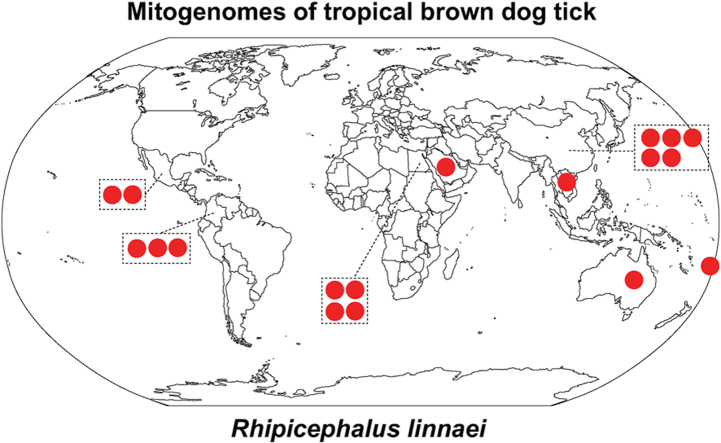

## 1 Introduction

The brown dog tick *Rhipicephalus sanguineus* (*sensu lato*) is a well-recognised tick parasitising dogs in the Americas (Dantas-Torres, 2010). Early studies investigating the distribution and genetic diversity laid the foundation for recognition of “southern” and “northern” lineages in South America (Moraes-Filho et al., 2011). The “southern” lineage was later recognised as the “temperate lineage” with the formal name *Rhipicephalus sanguineus* (Latreille, 1806) referred here to as *R. sanguineus* (*sensu stricto*) *via* a redescription of a neotype (Nava et al., 2018). The “northern” lineage or the “tropical lineage” was recently redescribed as *Rhipicephalus linnaei* (Audouin, 1826) using a neotype from Egypt (Šlapeta et al., 2022). Moraes-Filho et al. (2011) have genetically analysed brown dog ticks from Linares, Nuevo Leon State, Mexico, and used partial ∼460 bp fragment of the mitochondrial *16S* rRNA gene to assign them to the “northern” lineage or what is now recognised as *R. linnaei.* More recently, brown dog ticks were demonstrated on dogs and a single cat infested by nymphs of the tick species in the State of Puebla in Mexico, and molecularly identified as *R. linnaei* based on partial mitochondrial *16S* rRNA gene (Salceda-Sánchez et al., 2023). Finally, the presence of *R. linnaei* was demonstrated across 12 states in Mexico based on partial fragments of the mitochondrial *12S* rRNA and *16S* rRNA genes and morphological investigation (Almazán et al., 2023). The presence of *R. linnaei* was further demonstrated across the state of Morelos in Mexico using a partial fragment of the mitochondrial *16S* rRNA gene (Nieto-Cabrales et al., 2024). In North America, in particular the USA, both species *R. sanguineus* (*s.s*.) and *R. linnaei* are present (Jones et al., 2017). These two species predominate in distinct, overlapping geographies and their activity peaks at different times of the year (Grant et al., 2023). In Canada, brown dog ticks are only rarely detected most often linked to international travel (Myers et al., 2024). All of the North American studies utilised partial 340–370 bp fragment of the mitochondrial *12S* rRNA gene to determine the identity of *Rhipicephalus* species (Jones et al., 2017; Grant et al., 2023; Myers et al., 2024). Based on these results, the brown dog tick in Mexico can be considered to represent only *R. linnaei* and *R. sanguineus* (*s.s*.) is currently absent.

Despite the interest on the brown dog ticks, *R. sanguineus* (*s.l*.), and general acceptance of species (former lineages) identity determination based on partial mitochondrial sequences, there is currently no reference of a complete mitochondrial genome (mtDNA) also referred as ‘mitogenome’ from American specimen of *R. linnaei*. Mitogenomes enable comparison of the entire genome (∼14 kb) compared to small fragments (300–500 bp) and play a crucial role when comparing tick species and establishing robust taxonomy coupled with a morphological investigation (Burger et al., 2014; Kneubehl et al., 2022; Šlapeta et al., 2022, 2023; Barker et al., 2023).

This study aimed to provide a reference mitogenome sequence for the brown dog tick *R. linnaei* from America. To do so, ticks were collected from dogs in Mexico, whole genome sequencing was performed and complete mitogenomes of *R. linnaei* assembled. The assembled mitogenomes were compared to available mitogenomes of *R. sanguineus* (*s.l*.). The sequence analysis was enriched by the addition of new *Rhipicephalus* spp. from the Arabian Peninsula. Overall, this study demonstrates the presence of *R. linnaei* in the Americas, Asia, Africa, and Australia based on their complete mitogenomes.

## 2 Materials and methods

### 2.1 Available material and morphological identification

Ticks were collected from dogs in northern Mexico in 2023 (Ciudad Victoria, Tamaulipas State; 23.735000, −99.130833). In total, 10 adult ticks (5 males and 5 females) from Mexico were used; all representing the genus *Rhipicephalus*. In addition, 10 ticks from dogs from Eastern Province of Saudi Arabia were collected in 2019 (Al-Ahsa; 25.383333, 49.600000); seven ticks represented the genus *Rhipicephalus*, and three were *Hyalomma* spp. that were not processed further. Tick specimens were stored in individual tubes with 70% (v/w) ethanol. All ticks were observed under a stereo microscope (SMZ-2B, Nikon, Australia) and a digital microscope (VHX-6000, KEYENCE Inc., Japan), and identified using published keys and guides (Walker et al., 2000).

### 2.2 DNA isolation and amplification of the partial mitochondrial *cox*1 gene

DNA was isolated from ticks stored in 70% (v/w) ethanol as previously described by Šlapeta et al. (2022). Briefly, dried ticks with incisions in their idiosoma were subjected to total tick genomic DNA (gDNA) isolation with the Monarch Genomic DNA Purification Kit (New England Biolabs, Melbourne, Australia). Exoskeletons were retained and preserved in 70% (v/w) ethanol. Extracted gDNA was stored at −20 °C.

The ∼600 nucleotide (nt) fragment of the mitochondrial DNA (mtDNA) cytochrome *c* oxidase subunit 1 (*cox*1) gene was amplified using the primer set S0725/S0726, as described in Chandra et al. (2019) and Šlapeta et al. (2022). MyTaq™ Red Mix (Bioline, Meridian Bioscience, Melbourne, Australia) was used for DNA amplifications in 30 μl reactions, with 2 μl (approximately 0.5–10 ng) of template gDNA. All reactions included PCR-grade water (ddH_2_O) as a no-template control. The PCR reactions were performed in a T100™ Thermal Cycler (BioRad, South Granville, Australia), and the PCR products were sequenced at Macrogen Ltd. (South Korea).

### 2.3 Genome skimming of *Rhipicephalus* spp. and assembly of mitogenome from next-generation sequence data

The gDNA isolated from four adult *Rhipicephalus* spp. ticks was used for genome skimming *via* next-generation sequencing (NGS) using a NEBNext® DNA Library Prep Kit followed by NGS using 150 bp paired-end Illumina NovaSeq 6000 sequencing system at a depth of 3 Gb or 1 Gb of raw sequence data (Novogene, Singapore). To enrich our *Rhipicephalus* spp. data, published next-generation data from Colombia were used (Paez-Triana et al., 2023); the raw FastQ data for representative samples from two localities were retrieved from the project number PRJEB64029 at Sequence Read Archive (SRA) data in GenBank (https://www.ncbi.nlm.nih.gov/sra).

The data were analysed on Artemis HPC (Sydney Informatics Hub, The University of Sydney). The whole mtDNA (mitogenome) was assembled from FastQ data using the GetOrganelle v1.7.5.3 pipeline (Jin et al., 2020) (https://github.com/Kinggerm/GetOrganelle). The complete circular mtDNA were aligned with available complete mtDNA sequences from species of the *R. sanguineus* (*s.l*.) complex in CLC Main Workbench 23 (CLC bio, Qiagen, Clayton, Australia). Mitogenomes of *R. microplus* and *R. australis* served as the outgroup.

### 2.4 Mitogenome phylogenetic analysis

Evolutionary analysis by maximum likelihood (ML) and minimum evolution method (ME). This analysis involved 30 nucleotide sequences. There was a total of 15,029 positions in the final dataset. Evolutionary analyses were conducted in MEGA11 (Tamura et al., 2021). The evolutionary model was selected based on fitting 24 different nucleotide substitution models; the model with the lowest Bayesian Information Criterion (BIC) scores was considered to describe the substitution pattern. For the entire *Rhipicephalus* spp. mitogenome alignment, the best model was GTR (General Time Reversible model) + G (Gamma distribution) + I (model allowed for some sites to be evolutionarily invariable) with 67 parameters and BIC score 114032.785 (ln = −56580.937) and was used for ML analysis. For the alignment with *R. linnaei* mitogenomes, the best model was HKY (Hasegawa-Kishono-Yano model) + G (Gamma distribution) with 42 parameters and BIC score 57130.890 (ln = −28301.006) and was used for ML analysis. The ME evolutionary distances were computed using the Maximum Composite Likelihood method (Tamura et al., 2004). Bootstrap test was used to calculate the percentage of replicate trees in which the associated mitogenomes clustered together (1000 replicates for ME, 100 replicates for ML).

## 3 Results

Ticks from Mexico morphologically belonged to *R. sanguineus* (*s.l.*) and exhibited features congruent with *R. linnaei* (Šlapeta et al., 2022). Four ticks underwent DNA isolation with DNA concentration from 10.08 ng/μl to 0.42 ng/μl. Initial amplification of *cox*1 yielded sequences identical with those of *R. linnaei* and two of the DNA samples were processed for next-generation whole genome sequencing. Sequencing of DNA from the tick voucher JS6619 (A2) produced 25,273,822 raw 150 nt reads totalling 3.8 Gb of raw data (Q30 = 92.71%; GC = 47.30%). Sequencing of DNA from the tick voucher JS6621 (B2) produced 31,876,342 raw 150 nt reads totalling 4.8 Gb of raw data (Q30 = 92.58%; GC = 47.30%). The assembly of the 150 bp pair reads, yielded 14,710 bp (#JS6619) and 14,711 (#JS6621) bp long contigs that were complete and represented circular mtDNA. The overlapping mtDNA regions using both libraries were almost identical except for 7 single nucleotide differences and one gap. The circular mtDNA of *R. linnaei* from Mexico encodes 13 protein-coding genes, two rRNA genes and 22 tRNAs. The new mitogenomes were 99.9% identical with 20 and 21 nucleotide differences from a reference *R. linnaei* mitogenome (NC_060409). The previously obtained partial sequences for the mitochondrial *16S* rRNA gene (OR388555) by Nieto-Cabrales et al. (2024) from Mexican *R. linnaei* were 100% identical with the complete mtDNA obtained in this study as well as the reference mitogenome of *R. linnaei* (NC_060409). Similarly, mitochondrial *16S* rRNA gene sequence (GU553075) from Moraes-Filho et al. (2011) that represents the original “northern” lineage is 100% identical with the newly obtained mitogenome data for the *16S* rRNA gene.

Seven ticks from Saudi Arabia morphologically fitted the general description of *R. sanguineus* (*s.l.*) with some exhibiting features congruent with *R. linnaei* as well as those of *R. turanicus* specimens (Šlapeta et al., 2022, 2023). Ticks underwent DNA isolation with DNA concentration ranging from 2.64 ng/μl to 0.40 ng/μl. Initial amplification of the *cox*1 gene yielded sequences identical with those of *R. linnaei* (*n* = 6; PP882806-PP882807 and PP882809-PP882812) and *R. turanicus* (*n* = 1; PP882808); one DNA sample from each tick species was processed for next-generation whole genome sequencing. The six *cox*1 sequences related to *R. linnaei* formed a monophyletic clade with known *R. linnaei cox*1 sequences; five sequences clustered with those from Egypt including the neotype sequence and one (P2/22:3) clustered with the divergent Egyptian *R. linnaei cox*1 sequence (Šlapeta et al., 2022). Next-generation sequencing of DNA from the tick voucher #JS6361 (P2/22:3: *R. linnaei*) produced 7,162,948 raw 150 nt reads totalling 1.1 Gb of raw data (Q30 = 93.62%; GC = 44.88%). Sequencing of DNA from the tick voucher #JS6375 (P2/22:4: *R. turanicus*) produced 6,816,768 raw 150 nt reads totalling 1.0 Gb of raw data (Q30 = 91.44%; GC = 47.62%). The assembly of the 150 bp pair reads, yielded 14,717 bp (P2/22:3; JS6361) and 14,714 bp (P2/22:4; JS6375) long contigs that were complete and represented circular mtDNA encoding 13 protein-coding genes, two rRNA genes and 22 tRNAs. The new mitogenome from Saudi Arabia (P2/22:3; #JS6361) was 96.69% identical with the reference *R. linnaei* mitogenome (NC_060409), but it was 99.98% identical to the mitogenome of *R. linnaei* (P1/22:20–2) from Egypt (OM994391) (Šlapeta et al., 2022). The new mitogenome #JS6375 was 99.06% identical with the reference mitogenome of *R. turanicus* (NC_035946).

**Fig. 1:**
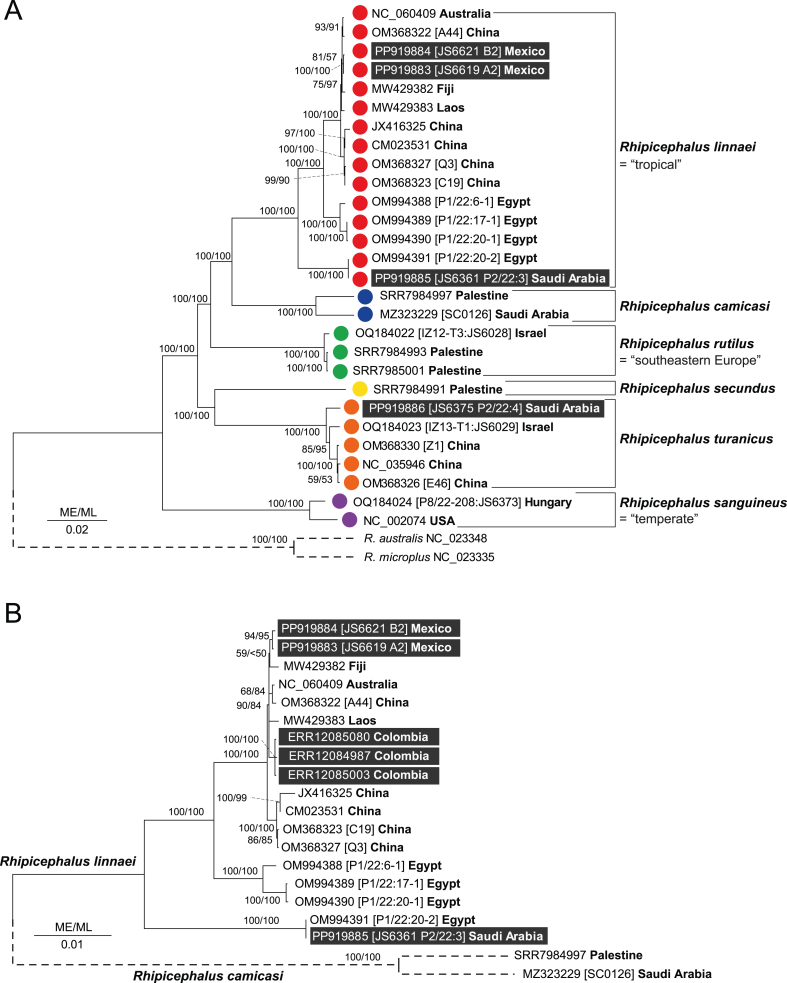
Phylogenetic relationships of *Rhipicephalus sanguineus* (*sensu lato*) based on complete mitogenomes. **A** Complete 30 mitogenomes of *R. sanguineus* (*s.l.*) were aligned and the tree was inferred from nucleotide data; there were a total of 15,029 positions in the final dataset. The optimal tree shown was inferred using the Minimum Evolution (ME) method. The tree was drawn to scale, with branch lengths in the same units as those of the evolutionary distances used to infer the phylogenetic tree. The evolutionary distances were computed using the Maximum Composite Likelihood method and are in the units of the number of base substitutions per site. For the ME tree, all ambiguous positions were removed for each sequence pair (pairwise deletion option). The percentage of replicate trees in which the associated taxa clustered together in the bootstrap test (1000 replicates for ME/100 replicates for ML [maximum likelihood]) are shown next to the branches. The species from which the mitogenome belongs to is indicated on the right and colour coded. **B** Complete 18 mitogenomes of *R. linnaei* were aligned together with two *R. camicasi* mitogenomes as outgroup. The tree was inferred from nucleotide data, there were a total of 14,755 positions in the final dataset. The optimal tree shown was inferred using the Minimum Evolution (ME) method. The tree was inferred as described above. Each mitogenome is identified by its GenBank accession number and voucher identifier together with the country where it was collected. The new mitogenomes from this study are shaded.

The four new mitogenome sequences (PP919883-PP919886) were appended to the mtDNA alignment of available *R. sanguineus* (*s.l*.) (Šlapeta et al., 2023). Both ML and ME reconstructed trees had identical topology for all species based on mitogenomes. Phylogenetic inference confirmed highly supported (100% bootstrap in ML and ME) monophyly of all new *R. linnaei* mitogenomes (Mexico: #JS6619, #JS6621; Saudi Arabia: #JS6361) with other *R. linnaei* sequences from Asia, Australia, and Africa. The Saudi Arabian *R. linnaei* mitogenome (P2/22:3; #JS6361) formed a monophyletic clade with the Egyptian mitogenome (OM994391) (Fig. 1A). The mitogenome of *R. turanicus* formed a highly supported clade that was sister to the mitogenome of *R. secundus*.

We have then generated mitogenomes from three randomly selected FastQ libraries from tick collected on dogs from Santander (*n* = 2; ERR12084987 and ERR12085080) and Casanare (*n* = 1; ERR12085003), Colombia. The assembly from the 150 bp pair reads, yielded 14,714 to 14,716 bp long contigs that represented circular mtDNAs encoding 13 protein-coding genes, two rRNA genes and 22 tRNAs. Appending the Colombian brown dog tick mitogenomes to the *Rhipicephalus* spp. alignment and inferring their phylogenetic position demonstrated that they unambiguously belong to the *R. linnaei* clade of mitogenomes with > 99.8% nucleotide sequence identity to reference *R. linnaei* as well as the Mexican reference mitogenomes (Fig. 1B).

## 4 Discussion

Brown dog ticks are the most common ectoparasite of domestic dogs in Mexico (Tinoco-Gracia et al., 2009), where its presence was documented over 100 years ago (Macías-Valadez, 1923). The brown dog tick was associated with a rickettsiosis outbreak that occurred in northern Mexico in 1947 (Álvarez-Hernández et al., 2017). Otherwise, infestations by the brown dog ticks were underestimated until 21st Century when outbreaks of rickettsiosis in the southwestern US state of Arizona and rural and suburban areas of Baja California in northwestern Mexico occurred in which the dog tick was demonstrated to be the vector (Demma et al., 2005; Parola et al., 2013).

**Fig. 2:**
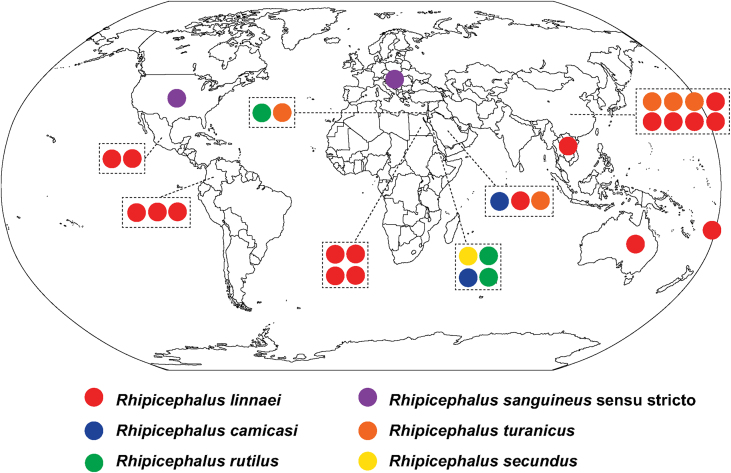
World map distribution of *Rhipicephalus sanguineus* (*sensu lato*) based on complete mitogenomes. Each mitogenome is colour coded (as in Fig. 1) based on species identification, each circle represents one mitogenome. Reference sequences for *R. linnaei* are now available from the Americas, Africa, Asia and Australia.

The brown dog ticks, *R. linnaei*, identified in this study were collected from dogs in Ciudad Victoria, Tamaulipas State, in northern Mexico, where brown dog ticks are seen throughout the year in untreated animals. Using these ticks, we have provided the reference mitogenome for the brown dog tick *R. linnaei* from the American continent complementing those from Asia, Africa and Australia (Jia et al., 2020; Šlapeta et al., 2021, 2022) (Fig. 2). Generating mitogenomes of ticks and other parasites is possible using genome skimming *via* next-generation sequencing (Jia et al., 2020; Šlapeta et al., 2021; Papaiakovou et al., 2023). The cost of genome skimming for ticks is comparable to the cost of PCR amplification and bi-directional Sanger sequencing of three or more amplicons, yet it generates all mitochondrial full coding sequences as well as both *12S* and *16S* rDNA gene sequences compared to a partial amplicon (Kneubehl et al., 2022; Šlapeta et al., 2022, 2023). Besides mitogenome, genome skimming enables screening for pathogens vectored by these ticks including bacteria, protozoans, fungi, nematodes, and viruses (Jia et al., 2020; Paez-Triana et al., 2023). Paez-Triana et al. (2023) generated shotgun next-generation sequence data from brown dog tick in Colombia that they considered the “tropical lineage” of *R. sanguineus* (*s.l.*) ticks in Colombia. Their focus was metagenomic identification of pathogens carried by these ticks, identifying *Anaplasma phagocytophilum*, *Francisella tularensis*, *Theileria equi* and the ubiquitous endosymbiont *Coxiella mudrowiae* (Paez-Triana et al., 2023). Here, we successfully used their data to complement our mitogenome from South-Central American countries for *R. linnaei.* The identity of *R. linnaei* from the Americas is now confirmed using complete mitogenomes from Mexico and Colombia.

During last century, literature referred to brown dog tick in Mexico as “*R. sanguineus*”. However, two genetic lineages of *R. sanguineus* in the American continent were recognised (Moraes-Filho et al., 2011), “temperate” or “southern” lineage now representing the *R. sanguineus* (*s.s.*), and “tropical” or “northern” lineage now representing *R. linnaei*. The distinction was supported with partial mitochondrial *12S* rRNA gene and *16S* rRNA gene sequences (Moraes-Filho et al., 2015; Grant et al., 2023). Aside from morphology and genetics, biological differences between *R. sanguineus* (*s.s*.) and *R. linnaei* in America exist. For instance, *R. sanguineus* (*s.s*.) undergo diapause, which helps it to survive in temperate regions of Argentina, Brazil, Uruguay, and the USA, whereas in *R. linnaei* diapause does not occur, thus explaining why *R. linnaei* is found parasitising dogs throughout the year in South-Central American countries (Labruna et al., 2017). This is in line with the findings of Galindo-Velasco et al. (2020) who studied the annual infestation pattern of *R. sanguineus* (*s.l*.) in the State of Colima in Mexico, finding that infestation occurs throughout the year with similar infestations from spring to winter.

The distribution of *R. linnaei* in Mexico was documented by Villarreal et al. (2018), reporting it from the southern Mexican State of Oaxaca through southern California and western Arizona in the USA. While the Villarreal et al. (2018) study was based on partial mtDNA sequences, these sequences match with *R. linnaei*. Therefore, morphological studies combined with molecular analysis demonstrate that *R. linnaei* is widely distributed in Mexico (Almazán et al., 2023; Nieto-Cabrales et al., 2024).

## 5 Conclusions

The species complex *R. sanguineus* is recognised to harbour multiple species; of these, two are known to be established in the Americas, *R. sanguineus* (*s.s*.) from South America and North America and *R. linnaei* that is the only species in Central America but extends to the warmer climates in South and North America as well (Jones et al., 2017; Díaz et al., 2018; Myers et al., 2024). Despite the reclassification of the “tropical lineage” of *R. sanguineus* (*s.l*.) as *R. linnaei*, this tick is still referred to as the “tropical lineage”. New mitogenome data from Mexico confirm that *R. linnaei* found in the tropics of the American continent is conspecific with the *R. linnaei* reference from Egypt. Based on these results, we conclude that tropical brown dog ticks from Mexico correspond to *R. linnaei*, making the term “tropical lineage” of *R. sanguineus* (*s.l*.) unnecessary in the Americas. Therefore, we propose using the complete mitogenome as the reference for American *R. linnaei*, even when only partial mitochondrial *cox*1, *12S* rRNA or *16S* rRNA genes are characterised.

## Funding

This research received no funding.

## Ethical approval

Dog sampling was approved by the Ethical and Bioethical Committee of the Faculty of Veterinary Medicine, Autonomous University of Tamaulipas, and sampling procedures were performed according to the guidelines of the Mexican Federal Law for Animal Health (DOF, 11-05-2022).

## CRediT authorship contribution statement

**Consuelo Almazán:** Project administration, Writing – review & editing. **Lorena Torres Rodríguez:** Project administration, Writing – review & editing. **Abdullah D. Alanazi:** Project administration, Writing – review & editing. **Jan Šlapeta:** Data curation, Resources, Supervision, Writing – review & editing.

## Declaration of competing interests

The authors declare that they have no known competing financial interests or personal relationships that could have influenced the outcomes of this study.

## Data Availability

Supplementary material, including sequence data, is available online at LabArchives (https://dx.doi.org/10.25833/vm6c-9a05). Sequence data reported in this study are available from GenBank SRA (PRJNA1113812). Partial *cox*1 from Saudia Arabian *Rhipicephalus* spp. was submitted to GenBank under accession numbers PP882806-PP882812 and mitogenomes of Mexican and Saudi Arabian *Rhipicephalus* spp. are under accession numbers PP919883-PP919886.
